# A New GRK2 Inhibitor for Heart Failure

**DOI:** 10.1016/j.jacbts.2024.12.006

**Published:** 2025-02-24

**Authors:** Chen Gao, Ningjing Song, Yibin Wang

**Affiliations:** a Department of Pharmacology, Physiology and Neurobiology, University of Cincinnati, School of Medicine, Cincinnati, Ohio, USA; b Signature Research Program in Cardiovascular and Metabolic Diseases, DukeNUS Medical School, Singapore; c National Heart Center of Singapore and National Heart Research Institute of Singapore, Singapore; d Department of Medicine, Duke University School of Medicine, North Carolina, USA

**Keywords:** cardiac pathophysiology, GPCR, GRK2, heart failure, LV function

**Figure:**
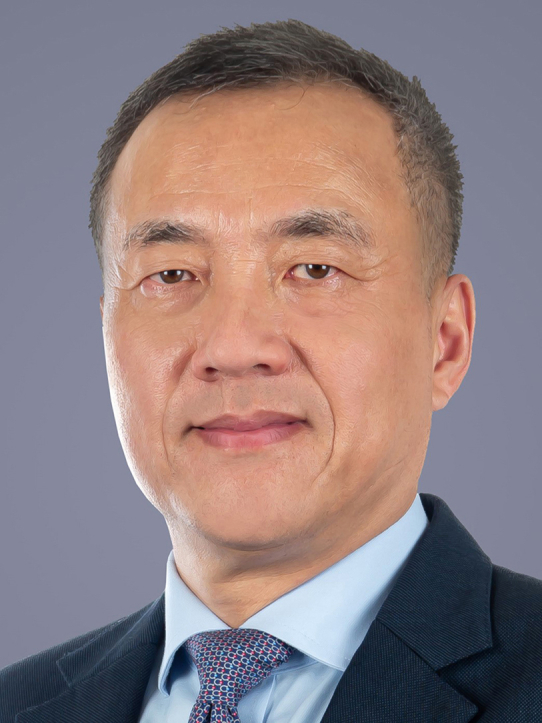

Ligand-induced adrenergic signaling controls cardiac contractility, metabolism, and growth through its cognitive G-Protein Coupled Receptors (GPCR), which are central to cardiac physiology and diseases. In human failing hearts as well as preclinical models of heart failure, desensitized and misregulated adrenergic signaling (or diminished and adverse adrenergic response) represents one of the most prominent molecular hallmarks in cardiomyocytes.^1^ Much of the clinical benefits observed from the treatment of adrenergic receptor antagonists (βAR blockers) can be attributed to resensitization of the adrenergic signaling in hearts. Therefore, preservation and restoration of adrenergic response has long been viewed as an effective therapeutic approach for heart failure. Like most GPCRs, βAR desensitization is triggered by serine/threonine phosphorylation in its protein C- terminus, carried out by a member of the GPCR associated kinases (GRKs) including GRK2.^2^ Thus, a logical step to prevent desensitization and restore GPCR sensitivity is to block GRK activity toward GPCR.^3^ In a seminal study by Koch et al^4^ almost 3 decades ago, the role of βAR kinase 1 in cardiac adrenergic receptor desensitization and cardiac dysfunctional as well as proof-of-concept evidence of targeted βAR kinase inhibition for heart failure were demonstrated, paving the way for subsequent development of βAR kinase inhibitors as heart failure therapy. However, earlier attempts utilizing a βAR kinase C-terminal peptide as the therapeutic agent encountered significant challenges in clinical implementation because of its reliance on viral vector-mediated gene transfer for effective expression in myocardium.^5^ Therefore, it was welcoming news and a significant step forward when Roy et al^6^ reported in this issue of *JACC: Basic to Translational Science* their preclinical results of a new small molecule-based GRK2 inhibitor for heart failure treatment.^6^

Although the scientific premise seems straightforward, developing a clinically effective inhibitor of GRK2 as heart failure therapy is a very challenging endeavor. The candidate molecules must possess high potency and specificity to the biological target while demonstrating good bioavailability and long-term safety. In 2012, Tesmer’s laboratory first identified that paroxetine, a U.S. Food and Drug Administration–approved serotonin reuptake inhibitor, could potently inhibit GRK activity through direct binding to its active site.^7^ This elegant study led to further optimization of paroxetine through rational design and the eventual identification of a novel GRK2 inhibitor, CCG258208 with superior properties over the parent molecule paroxetine in potency and specificity.^8^

Built upon this progress, Roy et al^7^ performed a comprehensive investigative new drug enabling study on CCG258208. At the cellular level, CCG258208 was able to elevate cyclic adenosine monophosphate to comparable levels as paroxetine but at a 10-fold lower dosage, confirming its potency as a GRK2 inhibitor. Pharmacokinetic analysis in mice showed its distribution in plasma, heart, and liver, but not in brain, a crucial property for heart failure therapy. Importantly, treatment with CCG258208 starting from 2 weeks post–myocardial infarction significantly preserved contractility and reduced pathological remodeling and infarct size in mice in a dose-dependent manner. Similarly, treatment of CCG258208 starting at 6 weeks post–pressure overload also halted functional deterioration, chamber dilation, hypertrophy, and fibrosis in mice. Finally, acute dobutamine responses based on change in contractile function were measured in swine with myocardial infarction–induced heart failure, before and after treatment with the CCG258208 or paroxetine. The results showed a significant improvement in response to βAR agonist by the CCG258208 over paroxetine, confirming the mechanism of action in a large animal model. All of these preclinical studies support the notion that the new GRK2 inhibitor possesses potent therapeutic potential for heart failure across different etiologies (Figure 1).

**Figure 1:**
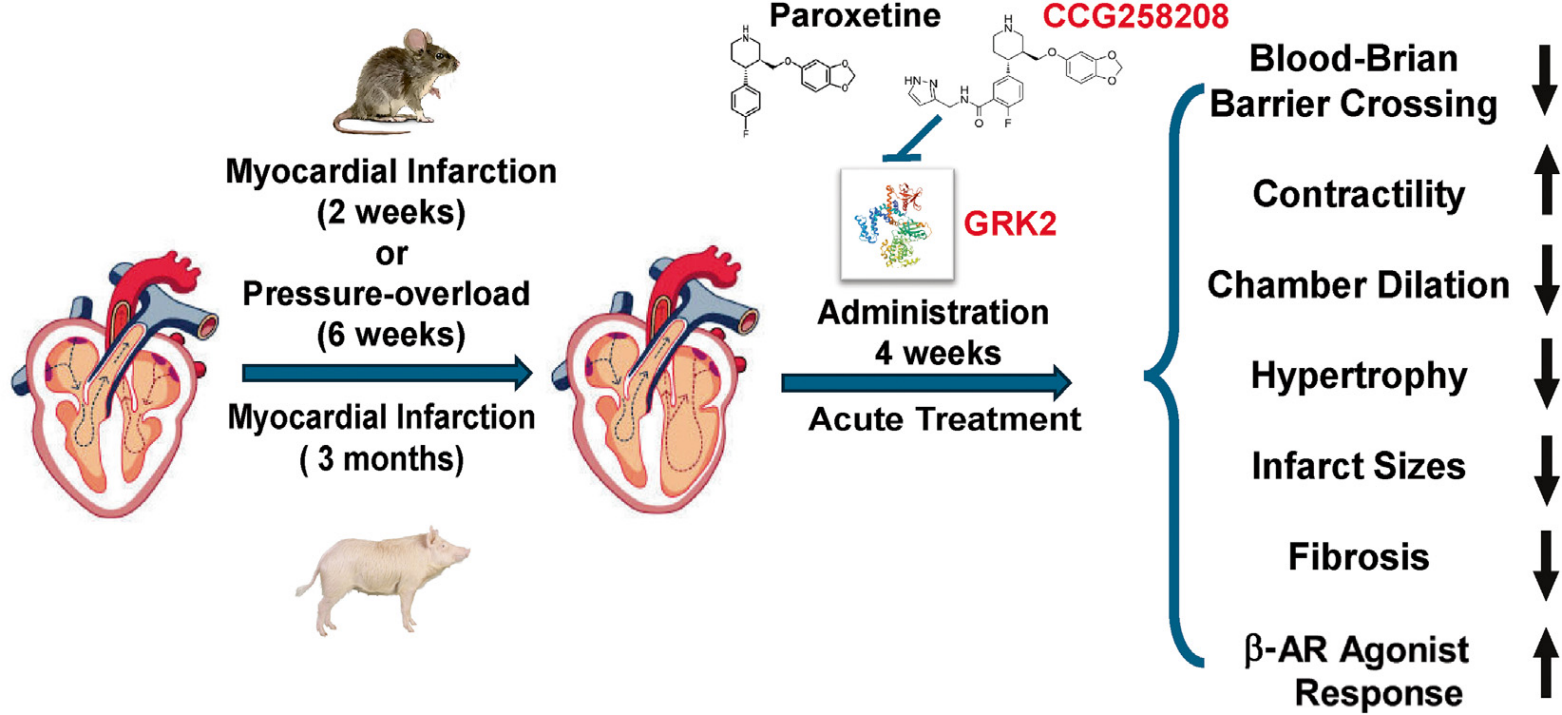
Schematic Illustration of In Vivo Demonstration of GRK2 Inhibition Using CCG258208 in Heart Failure Treatment

Although this study is encouraging, several aspects should be carefully deliberated in preparation for potential clinical applications. The long-term toxicity profile of CCG258208 remains to be further established. Despite the preferred tissue distribution profile, low penetration across the brain-blood barrier, and lack of noticeable adverse effects after 4 weeks of treatment in mice, more systematic evaluation would be required, including cardiac electrophysiology, blood chemistry parameters, and liver toxicity markers. Furthermore, the long-term efficacy in improvement of mortality would need to be demonstrated beyond the relatively short window of treatment reported in this study. A more challenging question for new therapeutics entering the heart failure space is whether the treatment can bring additional benefits or protection beyond the current standard care. Therefore, the therapeutic efficacy of this new GRK2 inhibitor must be benchmarked against the available beta-blockers. Considering the well-established noncanonical function of GRK2 independent from its negative modulation on G-protein coupled receptors, it is reasonable to be optimistic about the added protection from this new treatment over the standard care for heart failure patients. Given the known effect of GRK2 in mitochondrial function and cardiac metabolism,^9^^,^^10^ the therapeutic outcome for this inhibitor may reach beyond the classic heart failure populations caused by hypertension or ischemic injury, and be extended to heart failure associated with metabolic disorders, such as diabetic cardiomyopathy and heart failure with preserved ejection fraction. If successful, this would bring a new class of therapeutics into the current armory against various forms of heart failure. Such a prospect would certainly serve as yet another undisputed testimony and sweet reminder to the power of fundamental research on G-protein coupled receptor biology over the past century in drug development.

## Funding Support and Author Disclosures

This work is partially supported by PR191670 to Drs Gao and Wang.
